# Therapeutic potential of dithiocarbamate supported gold compounds

**DOI:** 10.1039/c9ra09682e

**Published:** 2020-01-16

**Authors:** Christian K. Adokoh

**Affiliations:** Department of Forensic Sciences, School of Biological Sciences, College of Agriculture and Natural Sciences, University of Cape Coast Cape Coast Ghana cadokoh@ucc.edu.gh kristattom@gmail.com

## Abstract

Chrysotherapy or aurotherapy, the use of gold as medicine, is two thousand years old. Hitherto, numerous diverse gold stabilizing ligands for instance vitamins, pyridine, phosphines, naphthylamine and xanthanes have been developed and their ‘chelating effect’ in addition to their anti-proliferative properties have been extensively studied. Recent advances in the field of bioinorganic chemistry have led to the design of biologically relevant metal complexes with appropriate fine-tuned ligands such as metallic conjugates of dithiocarbamates (DTCs). DTC compounds have been recognised to possess diverse applications and have demonstrated interesting biological properties. For instance, the chemoprotective and antitumour properties of gold metal ions and DTC compounds respectively, presents an innovative and effective approach to cancer management. This review presents therefore the therapeutic potential of DTC ligand systems as a support for gold compounds. The importance of dithiocarbamate supported gold compounds as potential therapeutic agents is highlighted with emphasis on the therapeutic potential of gold(iii) and gold(i) dithiocarbamate derivatives.

## Abstract 2

Chrysotherapy or aurotherapy, the use of gold as medicine, is two thousand years old.

## Graphical abstract

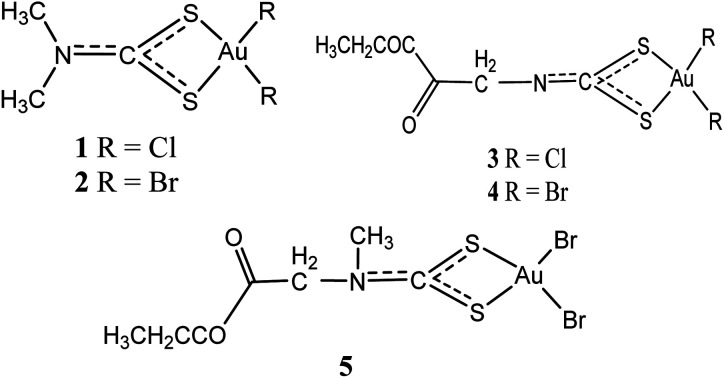

## 1 Introduction

New metal compounds are been synthesized each day with the aim of improving the cytotoxicity profile of anti-cancer drugs to achieve greater success in cancer treatment.^1^ In all these formulations, new ligand systems are developed and chelated to metal centres.^2^ The role of ligands in tuning the cytotoxic characteristics of complexes are of considerable significance. Metal complex lipophilicity and stability strongly depend on the nature of the ligand systems.^3^ Hence, a metal-based drug architecture and choice of ligand system provide significant characteristics that may control the toxicity, bioavailability, and specificity of metallodrug candidates.^4^ To this end, several ligand systems have been developed and dithiocarbamates have emerged as one of the ligand systems of choice for various applications in medicine such as for carbonic anhydrase (CA) inhibitors as well as important compound in cellular metabolism.^5^ DTC compounds and its metal complexes have the aptitude of modulating key proteins involved in biological processes such as apoptosis, transcription, oxidative stress and degradation.^6^ Coordinated dithiocarbamates are reported to possess potential chemoprotective function,^7^ treatment of bacterial and fungal infections, HIV-AIDS, and currently cancer.^8^ The effect on tumour cells is ascribed to their reactivity with copper in tumour cells to form complex, which inhibit proteasome and subsequently initiate tumour cell-specific apoptosis. Metal complexes formed by bidentate ligands which dithiocarbamates is an example, forms quite stable molecules. This is because of the so called “chelate effect” and the fact that the possibility of decomposition and ensuing loss of the dithiocarbamato ligand is impossible to occur.^8^ In addition, square planner complexes of dithiocarbamates yield more stable complexes due to coordination of additional S-donor moiety such as cysteine, methionine *etc.* The stability has been possible owing to *trans* arrangement of S-donor moiety to the –NCSS moiety resulting from the strong *trans*-influencing effect of the dithiocarbamato sulphur atoms. Consequently, further interactions of the metal centre with other thiol containing biomolecules likely to generate severe side effects, for example, liver, kidney toxicity (hepatotoxicity, nephrotoxicity respectively) *etc.* are prohibited.^8^

Taking into consideration the distinctive attributes and in-depth understanding of the biological properties and recognition phenomena involving dithiocarbamates and biological systems, several dithiocarbamates complexes, nanoparticles, and polymers are currently been developed for therapeutic applications and have shown interesting efficacies. The interest of these dithiocarbamato complexes is gradually growing not only because of their anticancer properties but rather their use for treatment of many other conditions such as: cocaine addiction,^9^ inflammation^10^ and viral infections.^11^ Different metals such as platinum, vanadium, ruthenium, gold, rhodium and many others have been implicated to possess therapeutic properties when conjugated with organic ligands^12,13^ Specifically, dithiocarbamates ligand systems have been of interest. The ligand is believed to have the properties to serve as a vehicle for transporting the metal to their intended target site^14^ and also has the tendency to reduce toxicity, improve solubility and biocompatibility.^15^

Dithiocarbamates supported gold compounds have been synthesized and exhibited various applications in medicine. Coordinated gold dithiocarbamates are reported to possess potential chemoprotective function and serve as a means of transporting metal to its active site. Significant improvement is required in the fabrication process from inexpensive to non-toxic materials and technique. This review highlights the importance of dithiocarbamates-based gold metal complexes, polymeric dithiocarbamates gold complexes and dithiocarbamates nano particulate systems towards the development of highly promising therapeutic drugs.

## 2 Dithiocarbamates gold compounds as therapeutic agent

The unique chemical properties of metal complex such as coordination of ligands in a three-dimensional configuration allow modification of clusters to be molded to well-defined molecular targets.^16^ This has been possible due to flexible chemical properties of metal ions, ligands and the complex itself. For example, transition metals reactivity properties such as charge variation, structure and bonding, metal–ligand interaction, Lewis acid properties, partially filled d shell and redox activity,^16–19^ offer scientists the broad spectrum of opportunities in modifying and fine-tuning during synthesis of novel compounds with anti-tumour potential.^20^ Additionally, metal ions and ligands possess wide range of properties such as coordination numbers and geometries, accessible redox states, thermodynamic and kinetic characteristics, which offer the medicinal chemist a wide range of re-activity that can be exploited to develop novel metal complexes of medicinal efficacy.^21^ The stabilization of a drug by coordination to the metal centres known as metal-drug synergism, enhances the activity of the organic molecule.^22^ Various diseases have been treated by metal complexes for centuries even before the introduction of Pt(ii)-based first anti-cancer agents.^23^ One notable example is the medicinal use of gold ‘chrysotherapy’. Among the oxidation states of Gold (−I, 0, I, II, III, IV and V), only gold 0, I and III are known to be the most stable and their compounds found to be highly active towards several forms of conditions. Gold compounds are emerging as a probable salutary agent in the treatment of arthritis, cancer, and HIV-AIDS. In view of this, several forms of gold compounds have been synthesized and tested for their therapeutic activities. In this section of the review, highlights of various forms of gold complexes used as therapeutic agents using dithiocarbamate as a ligand are described.

### 2.1. Therapeutic potential of dithiocarbamates gold(iii) derivatives

Gold(iii) compounds are assumed to act in the same manner as anticancer agent just as cisplatin and cisplatin-related anti-tumour drugs, due to their structural and electronic similitude.^24^ As such, these compounds are forthcoming as an innovative cluster of metal complexes with exceptional cytotoxic properties, and presently being assessed as probable anti-tumour agents. Ronconi *et al.* have pointed out that most dithiocarbamates gold(iii) derivatives have proved to be 1 to 4 magnitude more cytotoxic *in vitro* than the reference drug (cisplatin) and these compounds are able to overcome both intrinsic and acquired resistance to cisplatin.^24^ Even so, the development of gold(iii) complexes as therapeutic drugs have been hindered due to low stability under physiological conditions. This property remains the critical limitation in the drug development of this kind.^25,26^

**Fig. 1:**
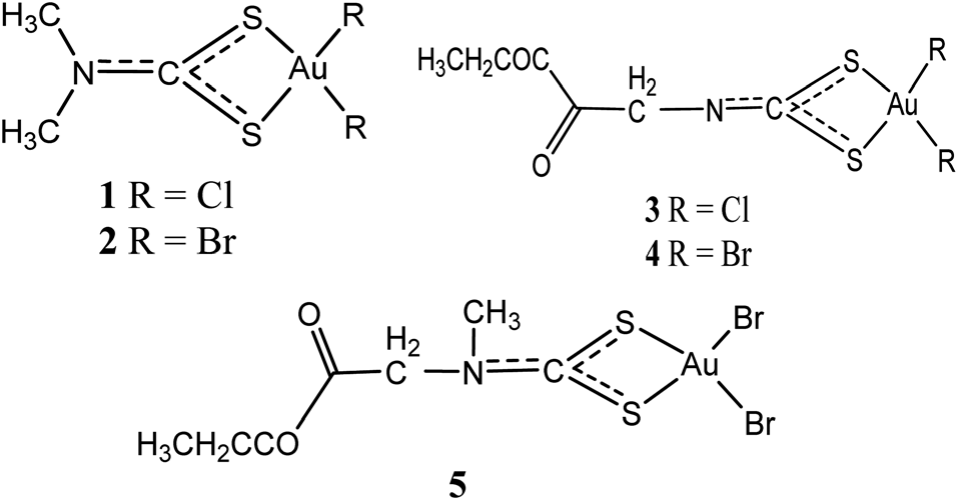
Chemical structures of gold(iii) dithiocarbamates derivatives 1–5.

In 2006, dithiocarbamates gold(iii) derivatives were developed particularly derivatives of *N*,*N*-dimethyldithiocarbamate and ethylsarcosinedithiocarbamate, such as Au(DMDT)Cl_2_ (1), Au(DMDT)Br_2_ (2), Au(ESDT)Cl_2_ (3), and Au(ESDT)Br_2_ (4) (Fig. 1). These compounds were found to inhibit cisplatin-induced nephrotoxicity and exhibited *in vitro* cytotoxicity toward a variety of human tumour cell lines.^6,27,28^ They were also found to exhibit 1–4 fold increase in potency compared to cisplatin as well as overcoming intrinsic and acquired cisplatin resistance.^6,29–31^ Furthermore, the result demonstrated that dithiocarbamates complexes act fast to inhibit RNA and DNA synthesis while demonstrating minimal cross-resistance with cisplatin, an indication of divergent mechanism of action.^25^ In another development, compound 2 was also found to potently inhibit the activity of a purified rabbit 20S proteasome and 26S proteasome in intact highly metastatic MDA-MB-231 breast cancer cells.^23^ Compound 2 significantly inhibited tumour growth when treated with MDA-MB-231 breast tumour–bearing nude mice cell and the mechanism of action has been associated with proteasome inhibition and massive apoptosis induction *in vivo*.^8^ Compounds 1 and 4 were also highly active against the androgen-resistant prostate cancer cell lines PC3 and DU145 than reference drug cisplatin, both impeding cell proliferation in a dose-dependent manner.^8^ Overall, compounds 1–4 are established to be more active than the reference drug cisplatin (*cis*-[PtCl_2_(NH_3_)_2_]). Compound 1 especially, exhibited cytotoxicity against cisplatin-resistant R-PC3 cells and the activity is similar to those induced on the parent cisplatin-sensitive PC3 cells. Normal cell cycle according to the authors was slightly affected due to early cell damage, portentous a dissimilar mechanism of action from clinically established platinum-based drugs.^8^ The authors also stated that the mitochondrial functions, promoting mitochondrial membrane permeabilization and Cyt-c release, stimulating ROS generation and strong inhibition of the activity of selenoenzyme TrxR which is overexpressed in prostate cancer associated with the onset of drug resistance was altered by these complexes. Furthermore, the complexes induce apoptosis, caspase activation, Bcl-2 down-regulation and Bax up-regulation, reduces the expression of the phosphorylated form of the EGFR, and inhibits PC3 cell migration.^8^*In vivo*, treatment of PC3 prostate tumour-bearing nude mice with compound 1 considerably subdued tumour growth, but triggering minimal systemic toxicity. Additionally, the same compounds 1–4 were found to inhibit thioredoxin reductase activity, generate free radicals, alter mitochondrial roles, and upsurge ERK1/2 phosphorylation.^32^ All these activities point to the fact that these compounds may be potential agents for cancer management and therefore the need for further investigation.

Similarly, an analog of compounds 3 and 4, ethylsarcosinedithiocarbamate [Au(iii)Br_2_(ESDT)] where ESDT = ethylsarcosinedithiocarbamate (5) has been investigated to put forth auspicious and exceptional anti-tumour activity *in vitro* overcoming both acquired and intrinsic resistance exhibited by different forms of tumours toward cisplatin^7^ In a studies using rodents on anti-tumour activity toward three transplantable murine tumour models, inhibition up to 80% tumour growth was recorded. The [Au(iii)Br_2_(ESDT)] (5) (Fig. 1) demonstrated low acute toxicity levels (lethal dose, LD_50_ = 30 mg kg^−1^) and reduced nephrotoxicity considerably.^7^

**Fig. 2:**
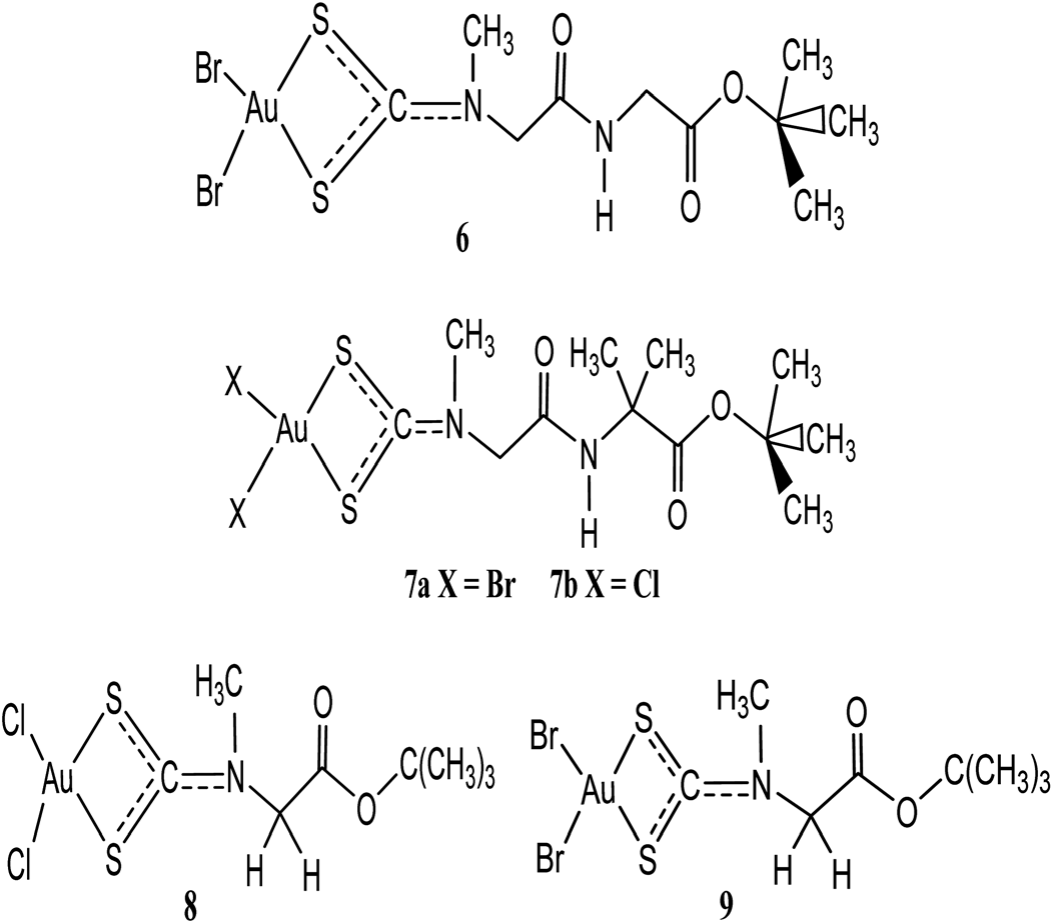
Chemical structures of gold(iii) dithiocarbamate derivatives 6–9.

Two anti-neoplastic agents developed *via* peptide transporters as carrier-mediated delivery vehicle: Au(iii) complexes, [Au(iii)Br_2_(dtc-Sar-Gly(AuD_6_)-O(*t*Bu))] (6) and [Au(iii)Br_2_(dtc-Sar-Aib(AuD_8_)-O(*t*Bu))] (7a) were synthesized and up regulated in some cancers (Fig. 2).^33^ Proteasome was the major target both *in vitro* and *in vivo* when they were tested on human MDA-MB-231 (cisplatin resistant) breast cancer cell cultures and xenografts. Treatment with these anti-neoplastic agents resulted in 53% inhibition of breast tumour growth in mice after 27 days compared to control. If only the most responsive mice were used, 85% growth inhibition was the result, with some animals showing tumour shrinkage after 13 days. These results demonstrate that gold(iii) peptidomimetics are suitable candidates as neoplastic agents and have been recommended to enter phase I clinical trials.^33^ Celegato *et al.* investigated novel gold(iii)-dithiocarbamato peptidomimetics, 7a–b (Fig. 2) compounds as preclinical activity of multiple-target in prostate cancer cells and xenografts.^33^ Compounds 7a–b exhibited higher cytotoxicity *in vitro* of about fivefold lower than the reference drug cisplatin. These compounds were also known to induce apoptosis and promote mitochondrial membrane permeabilization as well as stimulation of reactive oxygen species generation.^34^ In another development, the same gold(iii) compounds have also recorded significant cytotoxicity against cisplatin-resistant parent cell lines PC3-R and DU145-R ruling out cross-resistance phenomena.^34^ This is not surprising since in prostate cancer PC3 and DU145 cells peptide transporters are overexpressed to a good extent providing potential preferential uptake of selectively accumulate anti-cancer peptidomimetics. In this design, the authors targeted gold(iii) dithiocarbamato peptidomimetics recognition of the whole metal compound that, once recognized, peptidomimetics inside the tumour cell may transport and deliver to a specific target site to exert its anticancer activity without affecting healthy cells supported by the improved cellular uptake and minimization of unwanted side effects. The results was further sustained in that the compounds proved to hold back the ‘normal’ development of cancers *in vivo*, by inducing up to 70% tumour mass decrease in prostate cancer, together with negligible (or even no) organ toxicity.^34^

Two promising anticancer agents of *t*-butylsarcosinedithiocarbamato gold(iii) derivatives: [Au(iii)Cl_2_(dtc-Sar-O(*t*-Bu))] (8) and [Au(iii)Br_2_(dtc-Sar-O(*t*-Bu))] (9), with excellent cytotoxic activity (both *in vitro* and *in vivo*) and negligible, or no toxic side-effects have been developed by Ronconi and coworkers.^35,36^ These gold(iii) dithiocarbamate derivatives 8 and 9 were also known to interact with glutathione faster (within 60 min). Their *in vitro* cytotoxic activity toward human squamous cervical adenocarcinoma (HeLa), Hodgkin's lymphoma (L540), and histiocytic lymphoma (U937) cells showed IC_50_ values of low micro molar region (0.4–1.1 μM), lower than the reference drug cisplatin.^37^

Giovagnini and co-workers earlier described the synthesis, characterization, and the *in vitro* cytotoxic activity of methylsarcosinedithiocarbamate and its *S*-methyl ester derivatives. The [Au(MSDT)X_2_] (MSDT = methylsarcosinedithiocarbamate; X = Cl, Br) complexes exhibited dose-dependent growth inhibition of both HL60 and HeLa cells, with IC_50_ values higher or comparable to reference drug. The [Au(MSDT)X_2_] compounds was found to induce apoptosis in both cell lines (HL60 and HeLa).^24^

**Fig. 3:**
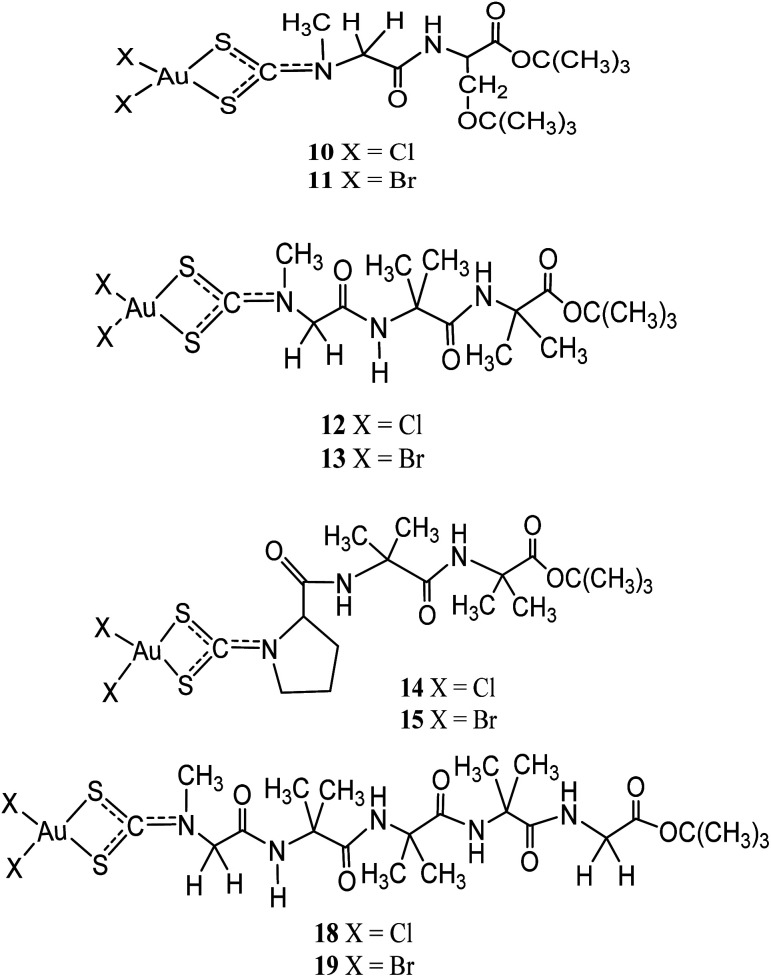
Chemical structures of gold(iii) dithiocarbamate peptidomimetics complexes 10–19.

Other than being used as therapeutics, gold(iii)-dithiocarbamate peptidomimetics compounds: [Au(iii)X_2_(dtc-Sar-L-Ser(*t*-Bu)-O(*t*-Bu))] (X = Br (10)/Cl (11)), [Au(iii)X_2_(dtc-Sar-Aib_2_-O(*t*-Bu))] (Sar = sarcosine, *N*-methylglycine), X= Br (12)/Cl (13); [Au(iii)X_2_(dtc-D,L-Pro-Aib_2_-O(*t*-Bu))] (X= Br (14)/Cl (15)), [Au(iii)X_2_(dtc-Sar-Aib_3_-O(*t*-Bu))] (X = Br (16)/Cl (17)), and [Au(iii)X_2_(-dtc-Sar-Aib_3_-Gly-OEt)] (X = Br (18)/Cl (19)) (Aib = “alpha”-aminoisobutyric acid, 2-methylalanine) were designed to precisely target two peptide transporters (PEPT1 and PEPT2) upregulated in several human tumour cells^38^ (Fig. 3). *In vitro* cytotoxicity studies by Kouodom and coworkers demonstrated that compound [Au^III^Cl_2_(dtc-D,L-Pro-Aib_2_-O(*t*-Bu))] (15) (Fig. 3) has strong activity against four human tumour cell lines (androgen receptor-negative prostate cancer PC3 cells, ovarian adenocarcinoma 2008 cells, the parent cisplatin-resistant C13 sub-clone, and Hodgkin's lymphoma L540 cell) with IC_50_ values lower than cisplatin.^38^ All the compounds have shown to promote overexpression of both peptide transporters PEPT1 and PEPT2 and also in either the normal genitourinary apparatus or healthy lymphocytes have not remained restricted demonstrating they're suitable as anticancer agents.^39,40^

**Fig. 4:**
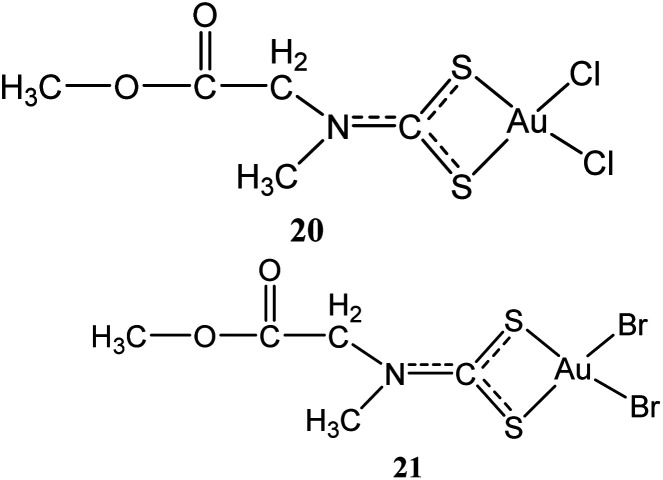
Chemical structures of gold(iii) methyl sarcosine dithiocarbamate derivatives 20–21.

Two other gold(iii) methyl sarcosine dithiocarbamate derivatives found active against myeloid leukemia: [Au(MSDT)Cl_2_] (dichloro[methyl-*N*-(dithiocarboxy-kS,kS′)-*N*-methylglicinato] gold(iii)) (20) and [Au(MSDT)Br_2_] (dibromo[methyl *N*-(dithiocarboxy-kS,kS′)-*N*-methylglicinato]gold(iii)) (21) (Fig. 4) were synthesized by Aldinucci and coworkers.^41^ These compounds combine cytostatic and apoptotic activity with reduced nephrotoxicity for the management of myeloid leukemia. The compounds can down-regulate Bcl-2 and upregulate Bax to induce cell death (Table 1).^41^ These novel gold(iii) dithiocarbamate derivatives probably present as potentially active drugs for the management of myeloid leukemia due to their cytostatic and apoptotic action coupled with reduced nephrotoxicity.

**Table 1:** Summary of biological activity of dithiocarbamates gold compounds

Compounds	*In vitro*/*in vivo* activity	Mechanism of action	Ref.
*N*,*N*-Dimethyldithiocarbamate gold(iii) complexes (1–2)	Inhibit cisplatin-induced nephrotoxicity and exhibited *in vitro* cytotoxicity toward a variety of human tumour cell lines. Compound 2 inhibited tumour growth when treated with MDA-MB-231 breast tumour-bearing nude mice cell	Proteasome inhibition and massive apoptosis induction *in vivo*	6, 27 and 28
Ethylsarcosinedithiocarbamate gold(iii) complexes (3–5)	Inhibit cisplatin-induced nephrotoxicity and exhibited *in vitro* cytotoxicity toward a variety of human tumour cell lines	Inhibit RNA and DNA synthesis while demonstrating minimal cross-resistance with cisplatin	6, 27 and 28
Gold(iii) dithiocarbamato peptidomimetics (6) [Au(iii)Br_2_(dtc-Sar-Gly(AuD_6_)-O(*t*Bu))]	Inhibit androgen-resistant prostate cancer PC3, DU145 cell lines and breast tumour in mice	Proteasome was the major target both *in vitro* and *in vivo*	33
Gold(iii)-dithiocarbamato peptidomimetics (7) [Au(iii)Br_2_(dtc-Sar-Aib(AuD_8_)-O(*t*Bu))]	Inhibit prostate cancer cells (PC3-R) and xenografts (DU145-R). Inhibit breast tumour in mice	Induce apoptosis and promote mitochondrial membrane permeabilization as well as stimulation of reactive oxygen species generation	34
*t*-Butylsarcosinedithiocarbamato gold(iii) derivatives (8) [Au(iii)Cl_2_(dtc-Sar-O(*t*-Bu))]	Cytotoxic toward human squamous cervical adenocarcinoma (HeLa), Hodgkin's lymphoma (L540), and histiocytic lymphoma (U937) cells	Interact with glutathione faster (within 60 min)	35 and 36
*t*-Butylsarcosinedithiocarbamato gold(iii) derivatives [Au(iii)Br_2_(dtc-Sar-O(*t*-Bu))] (9)	Cytotoxic toward human squamous cervical adenocarcinoma (HeLa), Hodgkin's lymphoma (L540), and histiocytic lymphoma (U937) cells	Interact with glutathione faster (within 60 min)	35 and 36
Gold(iii)-dithiocarbamate peptidomimetics compounds (10–19)	Inhibit androgen receptor-negative prostate cancer PC3 cells, ovarian adenocarcinoma 2008 cells, the parent cisplatin-resistant C13 sub-clone, and Hodgkin's lymphoma L540 cell	Target two peptide transporters (PEPT1 and PEPT2)	38–40
Dichloro[methyl *N*-(dithiocarboxy-kS,kS′)-*N*-methylglicinato]gold(iii)) (20) and dibromo[methyl-*N*-(dithiocarboxy-kS,kS′)-*N*-methylglicinato]gold(iii) (21)	Active against myeloid leukaemia	Act *via* cytostatic and apoptotic activity pathway by down-regulate Bcl-2 and up regulated Bax to induce cell death	41
Bipyridine gold(iii) dithiocarbamate-containing compounds	Cytotoxic toward prostate, breast, ovarian cancer cell lines and to Hodgkin lymphoma cells with IC_50_ values lesser than the standard drug cisplatin	Induce apoptosis, mitochondrial membrane depolarization, cytochrome-c release and caspase 9 activation	42
Triorganophosphinegold(i) dithiocarbamate (R_3_PAuS_2_CNR_2_)	Cytotoxic against seven human cancer cell lines namely: A498, renal cancer; MCF-7, estrogen receptor (ER)+/progesterone receptor (PgR)+; EVSA-T, estrogen receptor (ER)/progesterone receptor (PgR)−; H226, non-small cell lung cancer; GROV, ovarian cancer; Ml9 MEL, melanoma; and WIDR, colon cancer	Thioredoxin reductase (TrxR) inhibitor	43
Phosphinegold(i) dithiocarbamates: Et_3_PAuS_2_COEt (22), Et_3_PAuS_2_CNEt_2_ (23)	Induced anti-proliferative effects towards several human cancer cells	Subdue cytosolic and mitochondrial TrxR. Activate caspase-3 as well as inducing apoptosis	57–59
Pyrazolyl-1-dithiocarbamato-triphenylphosphinogold(i) (24), 3,5-dimethylpyrazolyl-1-dithiocarbamatotriphenylphosphinogold(i) (25) and indazolyl-1-dithiocarbamato-triphenylphosphinogold(i) (26)	Active against human cervical epithelioid carcinoma (HeLa) cells with tumour specificity (TS) ∼ 3.5	No mechanism of action has been reported yet	60 and 61
Bis-(pyrazolyl-1-dithiocarbamato)-bis(diphenylphosphino)alkyl dinuclear gold(i) (27), bis-(3,5-dimethylpyrazolyl-1-dithiocarbamato)-bis(diphenylphosphino) alkyl dinuclear gold(i) (28), bis-(indazolyl-1-dithiocarbamato)-bis-(diphenylphosphino)alkyl binuclear gold(i) (29)	Active against human cervical epithelioid carcinoma (HeLa) cells with IC_50_ values of 0.51–0.14 μM	No mechanism of action has been reported yet	60
Mononuclear *tert*-butyl dibenzyldithiocarbamate gold(i) compounds [*t*-Bu_3_PAuS_2_CN(C_7_H_7_)_2_] (30)	Cytotoxic against human cancer cell lines: HCT15, HeLa and A549 cell lines		61
Binuclear bis(diphenylphosphino)methane compounds: [(DPPM)Au_2_(S_2_CN(CH_3_)_2_)_2_] (31), [(DPPM)Au_2_(S_2_CN(C_2_H_5_)_2_)_2_] (32) [(DPPM)Au_2_(S_2_CN(C_7_H_7_)_2_)_2_] (33)	Cytotoxic against human cancer cell lines: HCT15, HeLa and A549 cell lines		61
Gold(i) dithiocarbamate compounds (34–36) (R_3_PAu[S_2_CN(iPr)CH_2_CH_2_OH], R = Ph, Cy and Et	Inhibit 25 strains of Gram-positive and Gram-negative bacteria pathogens, including the MRSA strain. Strong activity against *Staphylococcus aureus* (MRSA) and *Bacillus* sp.		62
Glycopolymer-DTC Gold(i) conjugate (37)	Demonstrate significant toxicity toward Hep G2, HEK 293T, MCF-7, and EMT-6 cells under hypoxic conditions		64
Tri-*tert*-butylphosphine and dialkyl dithiocarbamate gold(i) compounds [*t*-Bu_3_Pau(S_2_CNMe_2_)] and [*t*-Bu_3_Pau (S_2_CNEt_2_)]	Have strong cytotoxic activity against A549, MCF7 and HeLa human cancer cell lines		65 and 66
Phosphanegold(i) dithiocarbamate complexes (38–42) [Au(PR_3_)(S_2_CNR′_2_)], R = methyl, ethyl, isopropyl and R′ = methyl, ethyl	Active against two human cancer cell lines A549 and HepG2.	Induce distortion of DNA double helix indicating that gold(i) complexes target intracellular DNA *in vitro*	67
Statistical glyco-dithiocarbamate copolymer [p(GMAEDAdtc-*st*-GAEMA) and p(GMA-EDAdtc-*st*-LAEMA)] triphenylphosphine gold(i) gold nanoparticles	Glyconanoparticles and their Au(i)PPh_3_ conjugates inhibit MCF7 and HepG2 cells		63

Another novel bipyridine gold(iii) dithiocarbamate-containing compounds exerting compelling anticancer activity against cisplatin-resistant cancer cells independent of p53 status have been developed recently by Altaf and coworkers.^42^ These compounds are found to be highly cytotoxic to prostate, breast, ovarian cancer cell lines and to Hodgkin lymphoma cells with IC_50_ values lesser than the standard drug cisplatin. In another development these gold(iii) compounds were tested against panel of non-small cell lung cancer cell lines which showed prominent cytotoxic activity independent of p53 status. In this case the p53 was found to have been knocked out in the ovarian A2780 model.^42^ This promising outcome certainly calls for a further preclinical evaluation to test the clinical potential of these new gold(iii) compounds.

In alternative development, the gold(iii)-dithiocarbamate compound AuL12 (dibromo [ethyl-*N*-(dithiocarboxy-kS,kS′)-*N*-methylglycinate]gold(iii)) even though endowed with promising *in vitro*/*in vivo* anti-tumour activity, the toxicological profile has been poor in its soluble form and stability in physiological conditions.^43^ To overcome this, the complex was modified with natural oligosaccharide carriers to augment its water solubility and stability under physiological conditions.^44^ In this work, three types of α-cyclodextrins (CDs), namely β-CD, Me-β-CD and HP-β-CD were used to formulate aqueous solutions of AuL12.^45^ In particular, the HP-β-CD proved able to protect the AuL12 compound from degradation in phosphate buffer used to mimic physiological conditions. AuL12 inhibits Lys48 self-polyubiquitination reactions and does not interfere with Ub-E2 interactions but thwarts the Ub activation by E1. Even when the E1-ubiquitin thioester was not formed, the authors claim that MALDI-MS measurements were able to identify the non-covalent adduct E1-Ub. The researchers also realized that AuL12 prevents proteasome in a cell with a similar potency to that of bortezomib with a concentration of 7 μM able to entirely blocking ubiquitin chain growth under cell-free conditions. The mechanism of action relies on an energy-dependent uptake. Similarly, AuL12 was formulated with 1,2-distearoyl-*sn*-glycero-3-phosphoethanolamine-*N* [amino(polyethylene glycol)-2000] (DSPEPEG2000) and Pluronic® F127 (PF127) micelles and both micelle systems were found to be suitable vehicles for AuL12.^45,46^ Further addition of a bombesin peptide analogue or the octapeptide CCK8 to the micelle surface also gave the AuL12 nanomedicines cancer targeting properties, which lead to enhanced *in vitro* selective antiproliferative activity.^45,46^

Recently, Massai and colleagues investigated this same dibromo [ethyl-*N*-(dithiocarboxy-kS,kS′)-*N*-methylglycinate]gold(iii) (AuL12) as cysteine protease inhibitors.^47^ The authors asserted that gold(iii) dithiocarbamate (AuL12) triggered a dramatic inhibition of human cathepsins (B and L) and of *L. mexicana* cysteine protease CPB2.8DCTE at 20 μM. The gold(iii) complex was also potent inhibitors of human protozoan parasites growth (*L. infantum*, *T. cruzi*, *T. b. brucei*, *T. b. rhodesiense* and *P. falciparum*) with IC_50_ values in the sub-micromolar range and may be a prospective agent for pharmaceutical application as antiparasitic agents.^48^

### 2.2. Therapeutic potential of gold(i) dithiocarbamate derivatives

Gold(i) compounds are well-known therapeutic agent due to their anti-arthritic and anti-inflamatory properties.^49,50^ Conversely, the limited efficacy of the well-known gold(i) compound (*e.g.* Auranofin) as an anticancer drug has triggered development of several gold(i) compounds.^23,28,51–56^ The toxicity of the current chemotherapeutic agents have therefore limited their efficacy in the treatment of tumours and other related diseases. As a result, scientists have joined the expedition of developing novel targeted therapies in the quest of reducing toxicity while maximizing potency.^6^ Dithiocarbamate gold(i) complexes have therefore been developed by several researchers and their therapeutic activities investigated. The reason behind the renaissance and the development of gold(i) compounds as potential therapeutic agent arose probably due to the fact that most gold(iii) compounds are unstable under physiological conditions and in most cases converted to gold(i) which is thermodynamically more stable.^23^ In this section of the review, various forms of dithiocarbamate gold(i) complexes and their therapeutic potentials will be discussed.

In 2004, series of triorganophosphinegold(i) dithiocarbamate (R_3_PAuS_2_CNR_2_) compounds were synthesized and their cytotoxicity evaluated against seven human cancer cell lines namely: A498, renal cancer; MCF-7, estrogen receptor (ER)+/progesterone receptor (PgR)^+^; EVSA-T, estrogen receptor (ER)/progesterone receptor (PgR)^−^; H226, non-small cell lung cancer; GROV, ovarian cancer; Ml9 MEL, melanoma; and WIDR, colon cancer. The dithiocarbamates derivatives proved more potent than cisplatin in all cell lines screened, with Et_3_PAu(S:CNEt_2_) (ID_50_ = 12 ng mL^−1^) being the most active compound, but not as potent as taxol.^57,58^ Subsequent to this, gold(i) compounds have been confirmed heady thioredoxin reductase (TrxR) inhibitors *in vitro* in the nano molar range. TrxR, overexpressed in many tumour cells have been found to contribute to drug resistance, however, it has emerged as a new target for anticancer drugs. Gandin and colleagues based on this reason developed diethyldithiocarbamate phosphine gold(i) complexes 22 and 23 that subdued cytosolic and mitochondrial TrxR.^58^ When cancer cells were pre-treated with gold(i) compounds, inhibitory effect of the redox proteins was also observed intracellularly. These gold(i) compounds induced anti-proliferative effects towards several human cancer cells, some of which endowed with cisplatin or multidrug resistance.^58^ In addition, the compounds were able to activate caspase-3 as well as inducing apoptosis.^59^

**Fig. 5:**
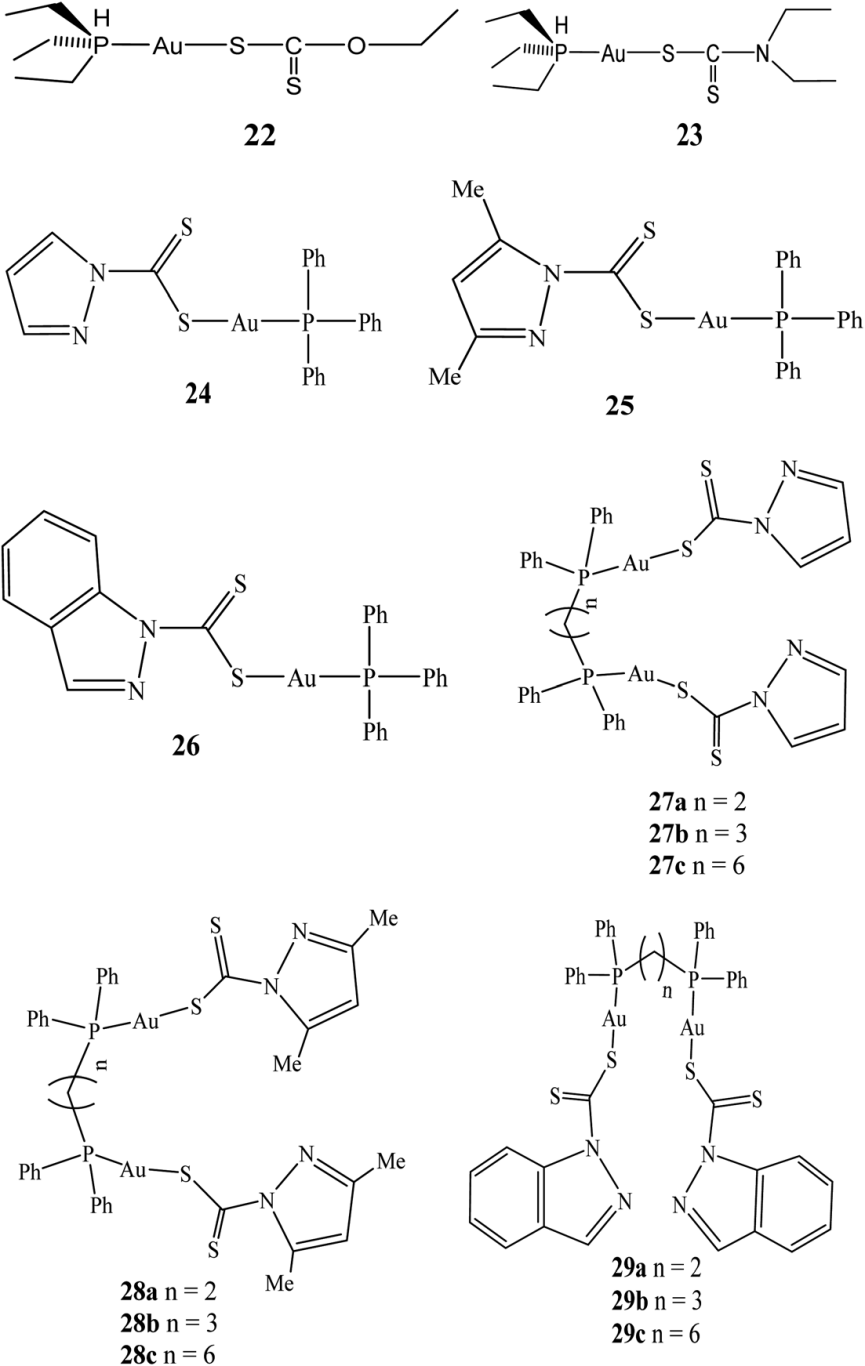
Chemical structures of phosphine gold(i) dithiocarbamates complexes 22–29.

In 2014, the first of triphenylphosphinegold(i) and dinuclear di(phosphino)alkylgold(i) dithiocarbamates compounds derived from heterocycles using triphenylphosphine, 1,2-bis(diphenylphosphino)ethane (dppe), 1,3-bis(diphenylphosphino)-propane (dppp), 1,6-bis(diphenylphosphino)hexane (dpph), and the pyrazol-1-yl- and indazol-1-yl-based dithiocarbamate ligands (dtcs) were reported by Keter and co-workers.^60^ The resulting triphenylphosphinegold(i) dithiocarbamates complexes: pyrazolyl-1-dithiocarbamato-triphenylphosphinogold(i) (24), 3,5-dimethylpyrazolyl-1-dithiocarbamatotriphenylphosphinogold(i) (25) and indazolyl-1-dithiocarbamato-triphenylphosphinogold(i) (26) (Fig. 5) were active against human cervical epithelioid carcinoma (HeLa) cells with tumour specificity (TS) ∼ 3.5.^60^ Similarly, dinuclear di(phosphino)alkylgold(i) dithiocarbamates complexes: bis-(pyrazolyl-1-dithiocarbamato)-bis(diphenylphosphino)alkyl dinuclear gold(i) (27), bis-(3,5-dimethylpyrazolyl-1-dithiocarbamato)-bis(diphenylphosphino)alkyl dinuclear gold(i) (28), bis-(indazolyl-1-dithiocarbamato)-bis-(diphenylphosphino)alkyl dinuclear gold(i) (29) were also synthesized (Fig. 5).

These dithiocarbamate gold(i) compounds demonstrated better activity against human cervical epithelioid compared to triphenylphosphinegold(i) dithiocarbamates compounds, but the most active compounds were found to be long alkyl chain bis-(pyrazolyl-1-dithiocarbamato)-bis(diphenylphosphino)hexane dinuclear gold(i) (27c) and bis-(3,5-dimethylpyrazolyl-1-dithiocarbamato)-bis(diphenylphosphino)hexane dinuclear gold(i) (28c), with IC_50_ values of 0.51 μM and 0.14 μM, respectively. Compounds 27c and 28c demonstrated selectivity toward HeLa cells more than they are toward normal cells, with selectivity's of 25.0 and 70.5 respectively.

Auxiliary tests, utilizing the 60-cell-line Developmental Therapeutics Program at the National Cancer Institute (U.S.A.), also revealed that 27c and 28c are active against nine other types of cancers *in vitro*.^61^ However, the results demonstrate that bis(diphosphines) alkanes with longer CH_2_ linkers (28c and 29c) appear to present better promise as anticancer agents than their shorter CH_2_-linker counterparts and could be further developed as an antineoplastic agent (Table 1).

**Fig. 6:**
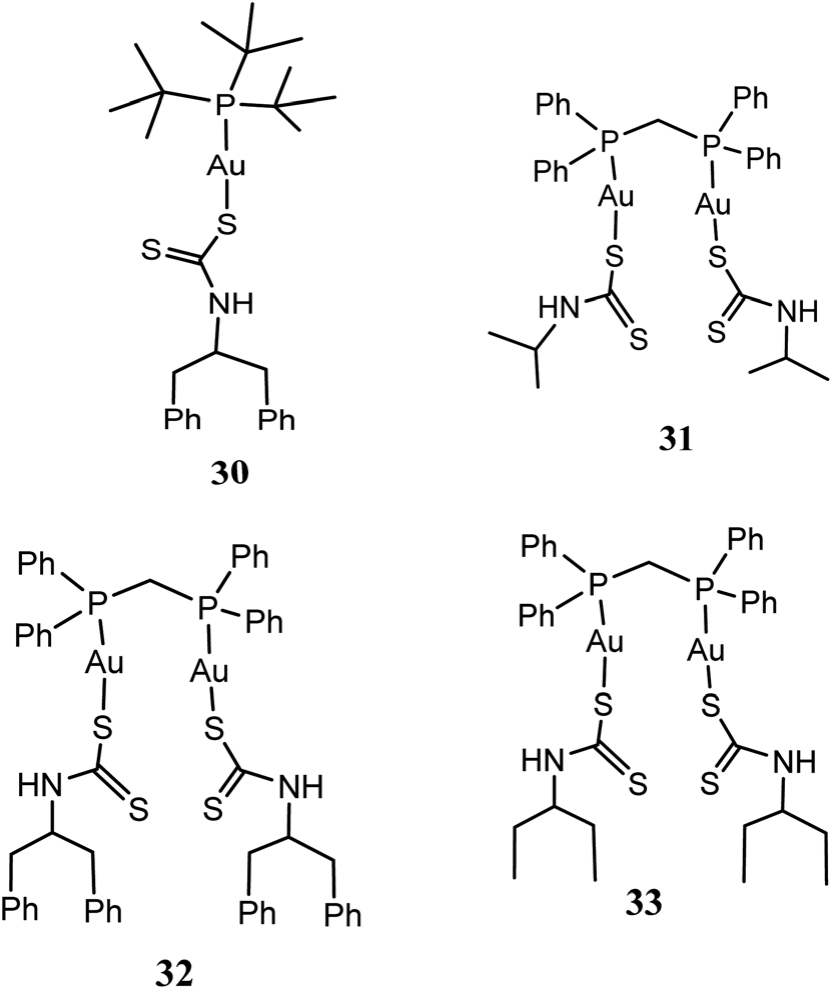
Chemical structures of phosphine gold(i) dithiocarbamate complexes 30–33.

New series of mononuclear [*t*-Bu_3_PAuS_2_CN(C_7_H_7_)_2_] (30), and binuclear [(DPPM)Au_2_(S_2_CN(CH_3_)_2_)_2_] (31), [(DPPM)Au_2_(S_2_CN(C_2_H_5_)_2_)_2_] (32) as well as [(DPPM)Au_2_(S_2_CN(C_7_H_7_)_2_)_2_] (33) [where DPPM = 1,1-bis(diphenylphosphino)methane, S_2_CN(CH_3_)_2_ = dimethyldithiocarbamate, S_2_CN(C_2_H_5_)_2_ = diethyldithiocarbamate and S_2_CN(C_7_H_7_)_2_ = dibenzyldithiocarbamate] gold(i) compounds have been synthesized and screened against three human cancer cell lines: HCT15, HeLa and A549 cell lines (Fig. 6).^61^ All the gold(i) compounds especially, 31 and 32 were established to have superior potent *in vitro* cytotoxic activity compare to cisplatin against HeLa, HCT15 and A549 cancer cell lines.^61^

**Fig. 7:**
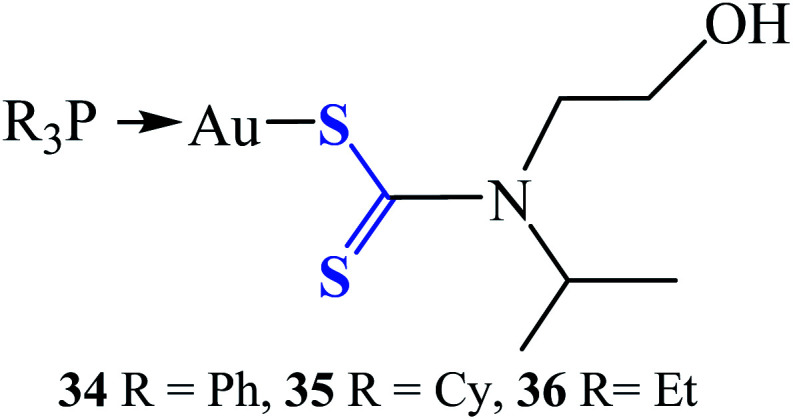
Chemical structures of phosphine gold(i) dithiocarbamates complexes.

Three gold(i) dithiocarbamate compounds (R_3_PAu[S_2_CN(iPr)CH_2_CH_2_OH] (Fig. 7), where R = Ph (34), Cy (35) and Et (36)) investigated *in vitro* for antibacterial and time-kill were found to be potent and possess differential activity against 25 strains Gram-positive and Gram-negative bacteria pathogens, including the MRSA strain. These bacterial pathogens are often multi-resistant to several classes of antibiotics and cause severe hospital-acquired and community-acquired infections.^62^ Compounds 34 and 35 were found to be specifically active against the tested Gram-positive bacteria, with minimum inhibition concentration (MIC) values ranging between 7.81 to 125 μg mL^−1^. Compound 36 was active against broad-spectrum of 24 strains of Gram-positive and Gram-negative bacteria, with MIC values ranging from 0.98 to 1000 μg mL^−1^. Particularly, strong activity against methicillin-resistant *Staphylococcus aureus* (MRSA) and Bacillus sp., was recorded and found to be as effective as the standard antibiotic ciprofloxacin with a very low MIC value of 0.98 μg mL^−1^.^62^ These compounds could be a prospective candidate as an antibacterial agent and as well as for the development of antibiotics.

**Fig. 8:**
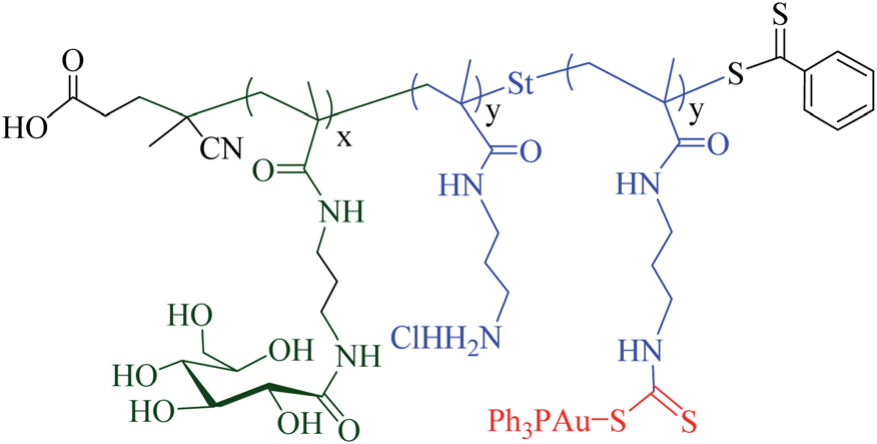
Chemical structures of glycopolymer-based gold(i) phosphines DTC-conjugates (37).

Polymeric vectors (*e.g.* carbohydrate polymeric vectors) are known to play an important role in cancer therapy. These vectors provide water solubility that facilitates nonspecific clearance, reduce toxicities and offer multivalency as well as targeting properties to the tumour site.^63^ Several metal polymeric vectors have been developed and studied extensively for their therapeutic properties. Ahmed and colleagues recently reported that glycopolymer-based gold(i) phosphines DTC-conjugates displayed higher accumulation and cytotoxicity in cancer cells under hypoxic conditions in comparison to the normoxic conditions (Fig. 8).^64^

Cytotoxic activity of two novel gold(i) compounds: [*t*-Bu_3_PAu(S_2_CNMe_2_)] and [*t*-Bu_3_PAu(S_2_CNEt_2_)] with S–Au–P fragment evaluated to have strong cytotoxic activity against A549, MCF7 and HeLa human cancer cell lines *in vitro* have been reported by Altaf and colleagues.^65,66^ The two new gold(i) compounds were developed using dimethyldithiocarbamate and diethyldithiocarbamate ligands and have shown tremendous cytotoxic activity against HeLa human cancer cell line. The result also demonstrates good inhibition and selectivity of both compounds against HeLa cell line which is very vital in drug design of target biomolecules.

**Fig. 9:**
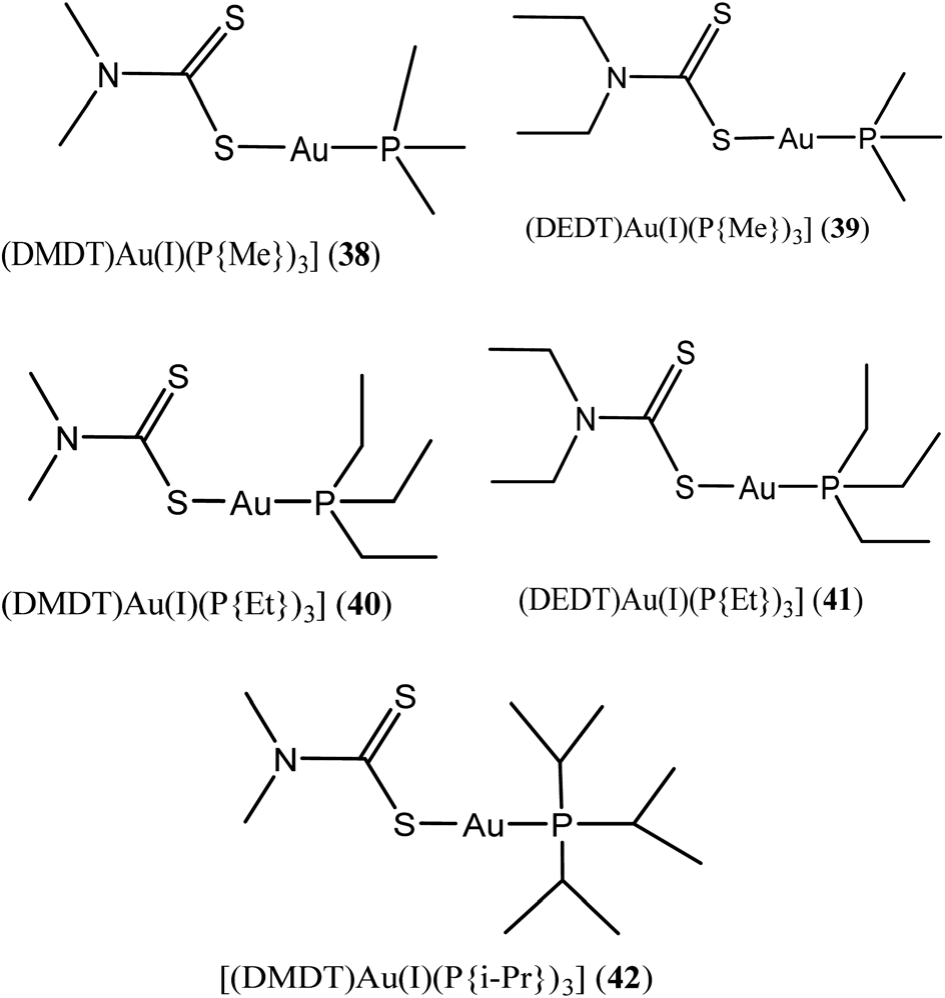
Chemical structures of novel phosphanegold(i) dithiocarbamates complexes 38–42.

Al-Jaroudi and colleagues, recently investigated novel phosphanegold(i) dithiocarbamate complexes with general formula [Au(PR_3_)(S_2_CNR′_2_)] 38–42 (where R = methyl, ethyl, isopropyl and R′ = methyl, ethyl) (Fig. 9) on two human cancer cell lines A549 and HepG2. The group posited that complexes 38–42 possess substantial potency of about 4 to 6-fold for A549 and 3 to 5-fold for HepG2 cell line than cisplatin.^67^ They also demonstrated *via* molecular docking studies that these complexes induce distortion of DNA double helix indicating that gold(i) complexes target intracellular DNA *in vitro*. The intrinsic binding constant (*K*_b_) values for 38–42 were also found to be very high (3.90 × 10^4^, 4.74 × 10^4^, 6.81 × 10^4^, 8.53 × 10^4^ and 2.48 × 10^4^ M respectively) which demonstrate their higher binding tendency.

### 2.3. Dithiocarbamates gold nanoparticles for therapeutic application

Another area which researchers have identified great prospect is the use of the dithiocarbamate gold nanoparticles as a plasmonic sensor in biological and environmental media. A dithiocarbamate gold nanoparticles (AuNPs) plasmonic sensor for simple and competitive detection of diafenthiuron has been developed with multifunctional groups of 4-hydroxy-6-methyl-3-nitro-2-pyridone-dithiocarbamate (HMNP-DTC) derivative.^48^ The multifunctional groups (OH, NO_2_ and CO) of HMNP-DTC-AuNPs exhibited strong ability to interact with diafenthiuron as a result of interaction between the HMNP-DTC-AuNPs and diafenthiuron to form a stable HMNP-DTC-AuNPs-diafenthiuron supramolecular network, which result in a red-shift in the SPR peak and a colour change from red to blue. The plasmonic sensor demonstrated high selectivity toward diafenthiuron with a detection limit of 7.1 nM, which is lower than the permissible limit of diafenthiuron according to the authors.^48^ The sensor, was successfully applied to detect diafenthiuron insecticide in environmental water and food samples with good recoveries.

**Fig. 10:**
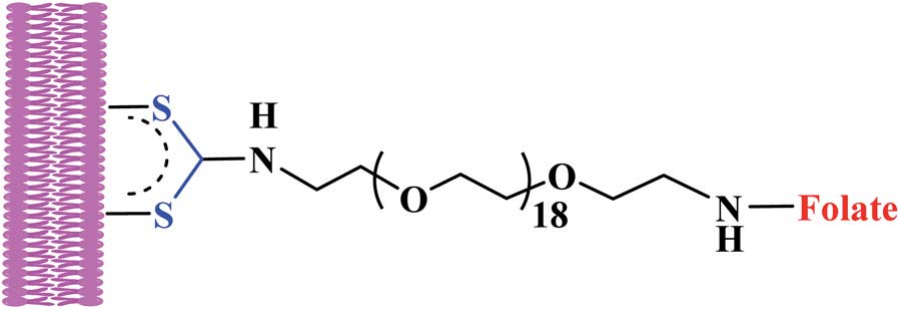
Folate-oligo-ethylene glycol dithiocarbamates gold nanorod.

In recent years, gold surface have been functionalized *via* DTCs ligand system as an alternative to thiols for medicinal purposes.^67–69^ The stability of DTCs in stabilizing gold nanoparticle is stronger compared to thiols to gold. DTCs nanomaterial's are also compatible with a wider range of environmental conditions.^68–72^ These properties according to many researchers are attributed to carbodithiolate (–CS_2_) groups having superior chemisorption properties with other molecules, as their interatomic S–S distances are nearly ideal for epitaxial adsorption onto Au surfaces.^70,73,74^ In line with the above properties, dithiocarbamates have been used extensively to stabilized gold nanorods (NR) for several applications instead of usual cetyltrimethylammonium bromide (CTAB), a cytotoxic surfactant used in nanorod synthesis (Fig. 10).

Recently, folate-conjugated gold nanorods (GNRs) synthesized *via* tethering onto GNRs surface by *in situ* DTC formation produced severe membrane damage upon near-infrared irradiation when surface of tumour cell was targeted.^68^ The investigation demonstrated rapid increase in intracellular calcium due to photo induced injury to the plasma membrane. In addition, formation of membrane blebs was also apparent due to disruption of the actin network.^68^ The same group investigated hyperthermic effects of dithiocarbamates gold nanorods on tumour cells and established that, nanorods rendered tumour cells highly susceptible to photothermal damage when irradiated at the nanorods' longitudinal plasmon resonance. In this case widespread generation of blebbing of the cell membrane was apparent at laser fluences as low as 44 W cm^−2^.^75^ This was possibly achieved due to nanorods directly targeting membranes of tumour cells *via in situ* dithiocarbamates formation as a result of robust attachment of ligands with high affinity for overexpressed cell-surface receptors.^75^ This demonstrates how dithiocarbamate moiety in the targeted drug delivery is important for tumours specificity. Dithiocarbamates GNRs have been investigated as contrast agents for biological imaging.^76^ According to Tong *et al.* 2009, dithiocarbamate GNRs can provide scattering contrast for dark field microscopy, or emit a strong two-photon luminescence due to plasmon-enhanced two-photon absorption in the cellular environment^76^ making nanorods attractive probes for *in vitro* and *in vivo* imaging. They also re-affirmed that GNRs can also efficiently transform optical energy into heat, and inflict localized damage to tumour cells. Additionally, laser-induced heating of nanorods can disrupt cell membrane integrity and homeostasis, resulting in Ca^2+^ influx and the de-polymerization of the intracellular actin network.^76^ The combination of plasmon resonant optical properties, intense local photothermal effects and robust surface chemistry of DTC render gold dithiocarbamates NRs as promising theragnostic agents.

Recently, synthesis of biomimetic biocompatible amino acid-dithiocarbamate (amino acid-DTC) gold nanoparticles (AuNPs) has been reported for surface-enhanced Raman scattering (SERS) imaging.^77^ The biomimetic amino acid-DTC AuNPs are excellent SERS contrast nanoprobe for cell imaging and possessed neglectful toxicity to human hepatoma cell. This property guaranteed the amino acid-DTC good biocompatibility for biomedical application. A strong SERS effect with an enhancement factor of 9.8 × 10^5^ was observed for biomimetic amino acid-DTC AuNPs for the sensing of Rhodamine 6G.^77^ This clearly demonstrate that the aptitude biomimetic amino acid-DTC AuNPs can be explored as an excellent SERS contrast nanoprobe for biomedical imaging. The amino acid-DTC mediated synthesis of the AuNPs may also possess great potential in bioengineering and biomedical imaging applications.

**Fig. 11:**
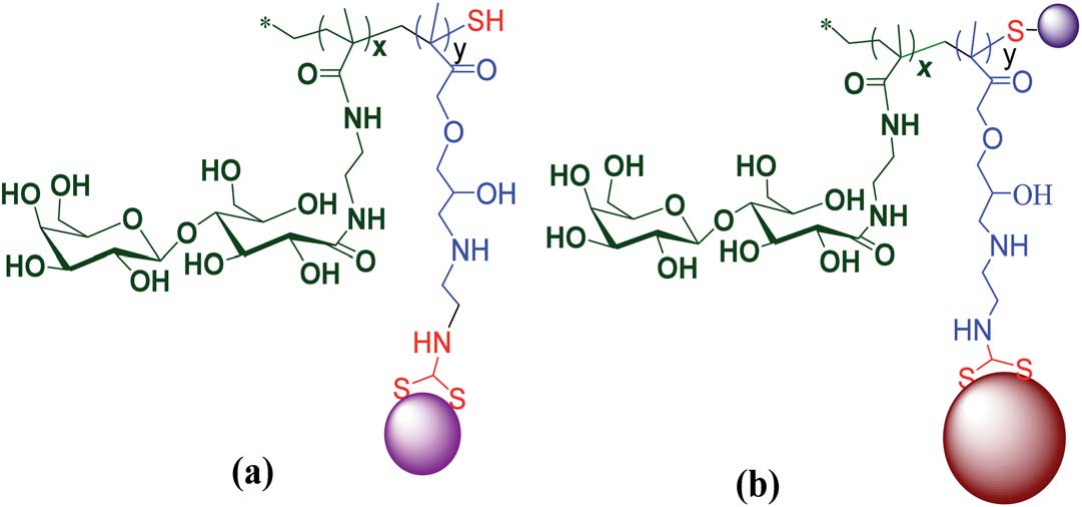
Chemical representations of (a) glyco dithiocarbamates gold nanoparticles (p(GMA-EDAdtc-*st*-LAEMA)AuNP) (b) glyco dithiocarbamate gold nanoparticles triphenylphosphine gold(i) conjugate (p(GMA-EDAdtc(AuPPh_3_)-*st*-LAEMA)AuNP).

Adokoh and colleagues synthesized well-defined statistical glyco-dithiocarbamate copolymer [p(GMAEDAdtc-*st*-GAEMA) and p(GMA-EDAdtc-*st*-LAEMA)], and their gold nanoparticles conjugated with triphenylphosphine gold(i) *via* reversible addition-fragmentation chain transfer polymerization (RAFT) polymerization^63^ (Fig. 11). A number of parameters such as molecular architectures, molecular weights and monomer ratios (carbohydrate to cationic segment) were varied and studied in detail for their cytotoxicity's and ability to act as non-viral gene delivery agents. The results revealed that statistical glyco-dithiocarbamates copolymer glyconanoparticles and their Au(i)PPh_3_ conjugates inhibit MCF7 and HepG2 cells. Galactose-functionalized glyconanoparticles gold(i) conjugates {P(GMAEDAdtc(AuPPh_3_)-*st*-LAEMA)AuNP} was the most cytotoxic agent to HepG2 cells (IC_50_ ∼ 4.13 ± 0.73 μg mL^−1^). Thus cytotoxicity of the nano material was four times more active in HepG2 cells compared to cisplatin and cytarabine. In this work, the overexpressed asialoglycoprotein (ASGPR) receptors was found to play an important role in the cytotoxicity profile, presumably due to the enhanced uptake^63^ presenting another strong case for dithiocarbamate gold compounds as potential therapeutic candidate.

### 2.4 Application of metallic DTC conjugates

Dithiocarbamates (DTCs) and their derivatives are widely used in diverse ways, but here, relevance will be given to medicinal important metal-chelating compounds. This class of compounds includes several drugs that have previously been approved for the treatment of various ailments, such as bacterial and fungal infections, as well as AIDS.^78,79^ During the World War II, DTC derivatives were first applied commercially as fungicides for treatment of crops, vegetables, seeds, and ornamental plants.^80^ In the household and rubber industries, the products (DTCs) have been used as animal repellents, biocides and accelerators respectively.^81,82^ Thiram, disulfiram, ziram, and ferbam are analogous dialkyl DTC used typically as pesticides. A monomeric DTC (Pyrrolidinedithiocarbamate (PDTC)) which contains a five member ring attached to its N atom has been used as metabolic inhibitor for cell physiological studies.^83,84^ Other forms of polymeric DTCs such as zineb, maneb, and mancozeb are commonly used in pre-harvest agricultural applications.^85^ As discussed above, the highly reactivity of DTCs anions to conjugate to thiol (SH) moiety and their ability to form metal chelates, gives them advantage to influence biological activities of different receptors (proteins, enzymes) and however, exert toxic effects. These characteristics have been exploited for their use in clinical applications.^86^

### 2.5 Molecular binding of gold to bioactive molecules

Proteins are essential in the formation of biological materials through their control of nucleation, growth, morphogenesis, phase organization, and distribution.^87,88^ Genetic engineering techniques (cell-surface display^89^ and phage display^90^) have led to the isolation of simple proteins with specific binding affinity to selected practical inorganics. The use of these proteins as building blocks has opened an avenue to the engineering of materials with novel properties. A recent example of this genetic approach is furnished by gold-binding proteins (GBPs). These proteins were selected for the ability to bind to gold in the presence of high salt concentrations, conditions under which other proteins do not exhibit gold binding.^89,91^ During the selection processes, investigation demonstrated that gold binding polypeptides bind more strongly to gold after the surface is treated with HF to remove surface impurities, indicating that the GBPs recognize the native gold surface rather than a partially removed contaminant. Additionally, GBPs preferentially bind gold over chromium, demonstrating substrate specificity.^89^ The GBP sequences contain multiple repeats of a 14–30 amino acid sequence.^89,91^ The repeating polypeptides retain their binding properties as part of other proteins (*e.g.* alkaline phosphatase) if they contain a sufficient number of repeats, and affinity for gold increases with the number of repeats. Interestingly, none of the GBPs sequences contains cysteine, which is known to form covalent bond with gold.^92^ Specifically, several researchers have demonstrated that dithiocarbamato derivatives of several transition metals, including Au(iii), Zn(ii) and Cu(ii), act as potent proteasome inhibitors in tumour cells.^93–96^ Their studies suggests that cellular proteasome is a molecular target for these DTC conjugated metal complexes.

## 3 Conclusion

This review has focused mainly on the therapeutic potential of dithiocarbamates gold compounds. The advent of chrysotherapy has brought about diverse architectures of gold compounds that have been well exploited for various applications ranging from therapeutics to drug delivery development. Despite the progress made thus far, several concerns still need to be addressed before the application of these gold compounds in clinics. Toxicity issues of these materials remain, perhaps, the most crucial that needs to be addressed. Gold(iii) compounds which have been found very active even more than current neoplastic agents cannot enter into clinical trials due to the issues of toxicity. It is therefore imperative to note that, the use of dithiocarbamates as carrier and ligand for gold compounds is promising and need to be probed further. Dithiocarbamates with their high water solubility and good stability in biological systems provide the outstanding potential for future biomedical applications. They can be considered as molecular carriers for the targeting, intracellular trafficking and delivery of biomolecules (proteins, peptides, drugs, genes). However, a better knowledge of how these dithiocarbamates gold(i) complexes, polymeric systems, and their nano particles interact with biological systems is required.

Diverse metals, for instance, platinum, vanadium ruthenium, gold, and rhodium, among many others, have been identified to possess medicinal properties when coordinated to organic ligands. The ligand is believed to serve as a delivery tool for conveying the metal to its active site and also has the tendency of reducing toxicity, improving solubility and biocompatibility. Coordination of metals to bioactive molecules could result in the formation of novel complexes or substrates that may possess greater activity/healing effect compare to original molecules. This is achievable since there may be protection of metal compounds from the normal metabolic pathways and slow release mechanisms. Ultimately, these formulations require new ligand systems development, and chelate to metal centres. The role of the ligands in tuning the cytotoxic characteristics of the metal compound is of considerable importance. Hence, the ligand choice or design for metal-based drugs imparts desirable features that may influence the toxicity, bioavailability, and the specificity of a metallodrug candidate. This requires carefully modification and development of ligands systems which strictly depends on type of target and treatment one want to repair. The metal compound lipophilicity and stability strongly depend on the nature of the ligand systems.

The objective in developing metal-based anticancer agents which are capable of incapacitating the problems of clinically used drugs while maintaining their efficacy has attracted attention all over the world. A widely used chemotherapeutic agent (*e.g.* Cisplatin) presenting extensive efficiency in the direction of solid tumours, and it remains the most widely endorsed anticancer drug, yet, the major obstacles for wider clinical application of these drugs have been severe toxicity, tumour resistance, and poor oral bioavailability. In an attempt to improve the side effect of these chemotherapeutic agents, gold compounds were tagged to replace the existing toxic one (platinum compounds) owing to their structural resemblance. Gold(iii) is iso-electronic with the d^8^ system and isostructural with platinum(ii)^97–101^ and the square planar gold(iii) complexes was identified as the probable replacement to possess potential anticancer activity. Indeed, these compounds have demonstrated stability under biological environments and also demonstrated considerable *in vitro* and *in vivo* anticancer activity which has led to influx of first-hand gold(iii) complexes since the commencement of 21^st^ century.^8,23,33,35^ Even though gold compounds have shown excellent anti-cancer properties, several glitches have hindered their development for clinical use. Issues such as severe toxicity to normal tissue or cells, intrinsic and acquired resistance to the treatment and bioavailability are some of the setbacks. One gold oxidation state which has proven to be highly active is gold(iii) compound, but its activity has been mired due to its instability state and reduce to gold(i) in solution or biological medium easily. However, the strategy has been coordinating metals centres to ligand systems capable of stabilizing it and offering other properties such as chemoprotective property. Further fine-tuning of the compound properties is also desirable for the specific biomedical application. In this case, one ligand system which has proven to perform these functions is dithiocarbamates. The excellent properties of this ligand system are that dithiocarbamates are bi-dentate chelating ligands and, upon coordination to a metal ion centre, the resulting compounds are expected to be quite stable owing to the ostensible ‘‘chelate effect’’. Stabilization of metals ion with dithiocarbamates results in a wide range of oxidation states, due to the presence of soft dithiocarbamates and resonance forms of hard thioureide. The thioureide ensue from the delocalization of nitrogen lone pair onto the sulphur, and their complexes may have a rich electrochemistry. The potential break down and the ensuing loss of dithiocarbamato ligand are improbable to take place. More importantly, the presence of the chelating dithiocarbamates is expected to create additional S-donor ligands interaction *trans* to the –NCSS moiety which is less favourable due to the rather strong *trans*-influencing effect on the dithiocarbamato sulphur atoms.^34^ These properties possibly may avert auxiliary interactions of the metal centre with other thiol containing biomolecules whose inhibition is generally allied with severe side-effects, such as kidney toxicity (nephrotoxicity). Dithiocarbamates are easily prepared from primary or secondary amines and exhibit good solubility in water or organic solvents depending upon the nature of the cation. As a result, dithiocarbamates ligands become better-leaving groups than chloride ions and may facilitate the availability of gold atom to interact with cancer cells to inhibit their growth *in vitro*. The major drug developmental change especially, in the field of anti-cancer therapy has been moving away from cytotoxic to a molecularly targeted agent. In this perspective, nano medicine has introduced new prospect in the cancer therapy. In this case, potential organometallic drugs can selectively be delivered to cancer cells leading to reduce toxicity. Dithiocarbamates ligand system has been used in this respect for drug targeting leading to enhanced cytotoxicity.

## Abbreviations

DTC: Dithiocarbamates
CA: Carbonic anhydrase
DMDT: *N*,*N*-Dimethyldithiocarbamate
ESDT: Ethylsarcosinedithiocarbamate
GBPs: Gold-binding proteins
HeLa: Henrietta Lacks
L540: Hodgkin's lymphoma
U937: Histiocytic lymphoma
MSDT: Methylsarcosinedithiocarbamate
CDs: α-Cyclodextrins
AuL12: Dibromo [ethyl-*N*-(dithiocarboxy-kS,kS′)-*N*-methylglycinate]gold(iii)
DSPEPEG2000: 1,2-Distearoyl-*sn*-glycero-3-phosphoethanolamine-*N* [amino(polyethylene glycol)-2000]
PF127: Pluronic® F127
R_3_PAuS_2_CNR_2_: Triorganophosphinegold(i) dithiocarbamate
TrxR: Thioredoxin reductase
GR: Glutathione reductase
GPx: Glutathione peroxidase
PDTC: Pyrrolidinedithiocarbamate
TS: Tumour specificity
DPPM: 1,1-Bis(diphenylphosphino)methane
S_2_CN(CH_3_)_2_: Dimethyldithiocarbamate,
S_2_CN(C_2_H_5_)_2_: Diethyldithiocarbamate
S_2_CN(C_7_H_7_)_2_: Dibenzyldithiocarbamate gold(i)
MIC: Minimum inhibition concentration
MRSA: *Methicillin-resistance Staphylococcus aureus*
HMNP-DTC: 4-Hydroxy-6-methyl-3-nitro-2-pyridonedithiocarbamate
–CS_2_: Carbodithiolate
CTAB: Cetyltrimethylammonium bromide
NR: Gold nanorod
GNRs: Folate-conjugated gold nanorods
AuNPs: Gold nanoparticles
SERS: Surface-enhanced Raman scattering
RAFT: Reversible addition-fragmentation chain transfer polymerization
ASGPR: Asialoglycoprotein

## Conflicts of interest

There are no conflicts to declare.

## Supplementary Material
